# Dr. Ratan Chandra Kar: The Journey of the Jarawa Doctor

**DOI:** 10.7759/cureus.64817

**Published:** 2024-07-18

**Authors:** Mitul Saha, Sonali G Choudhari, Swarupa Chakole, Sana Ahmed

**Affiliations:** 1 Department of Community Medicine, Jawaharlal Nehru Medical College, Datta Meghe Institute of Higher Education and Research, Wardha, IND; 2 Department of Community Medicine, Jawaharlal Nehru Medical College, School of Epidemiology and Public Health, Datta Meghe Institute of Higher Education and Research, Wardha, IND

**Keywords:** jarawa tribe, historical vignette, padma shri, andaman islands of india, indigenous tribe, tribal health

## Abstract

A distinguished physician Dr. Ratan Chandra Kar, born in 1954 in West Bengal, India, is known for his pivotal role in providing healthcare to the Jarawa tribe of the Andaman Islands. He began his service toward the Jarawa tribes in 1998, notably combating a devastating measles outbreak in 1999 that threatened the tribe's existence. Overcoming initial distrust, Dr. Kar earned the tribe's confidence through cultural respect and medical expertise, treating over a hundred patients at the peak of the epidemic. He had established a dedicated Jarawa Ward at Kadamtala Hospital, integrating their traditional practices with modern medicine. For his dedication, Dr. Kar received the Padma Shri in 2023, for contributing significantly to the tribe's growth from 255 to 260 individuals in 1998 to over 560 today. His work stands as a testament to the importance of culturally sensitive healthcare in preserving vulnerable indigenous communities.

## Introduction and background

**Figure 1:**
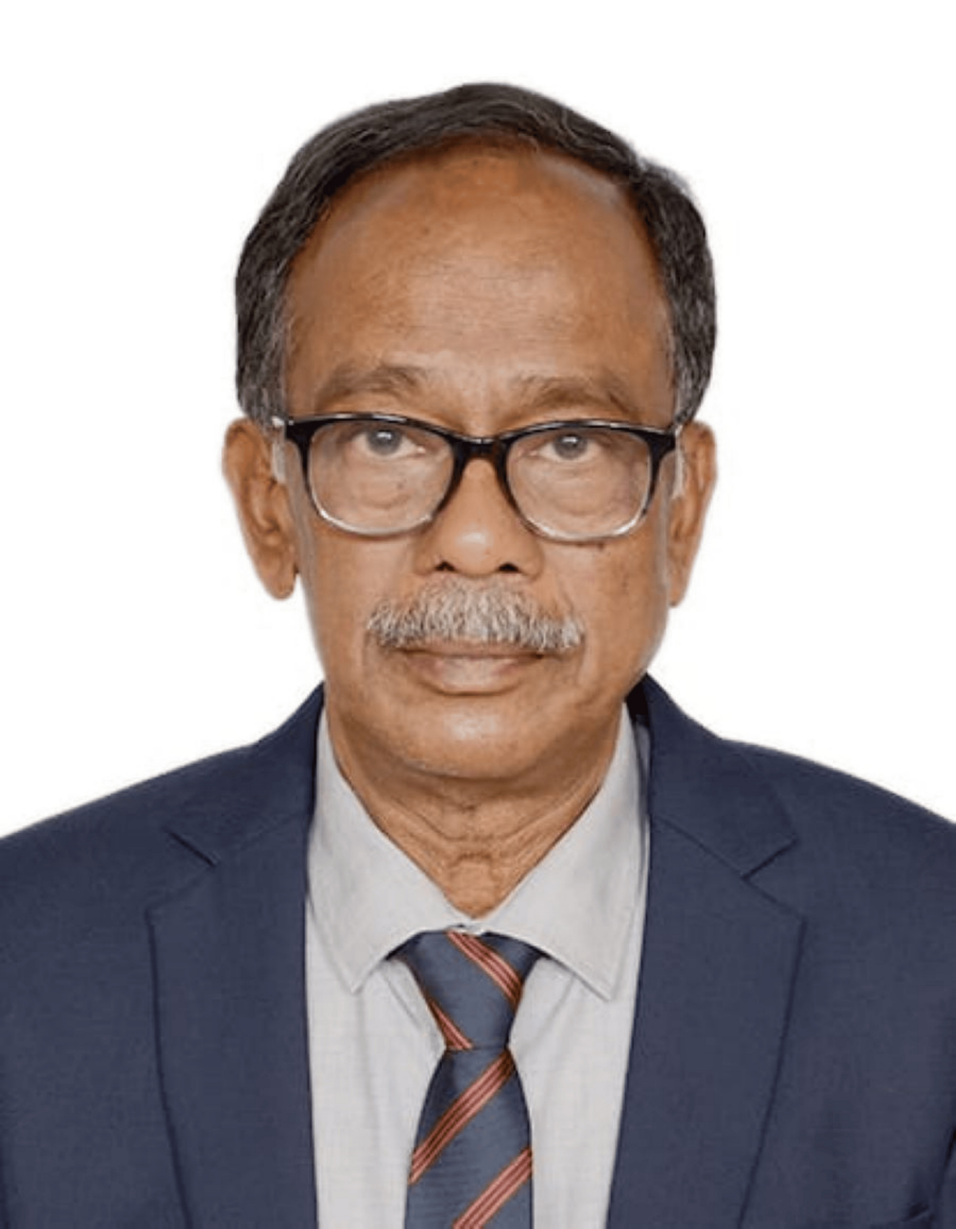
Dr. Ratan Chandra Kar Authors have received the permission from Dr. Kar to publish this photograph

Dr. Ratan Chandra Kar (Figure 1) is an Indian health official and physician [1]. He was born on May 4, 1954, in the Ghatal hamlet of West Bengal [2]. He is a 1974 Bachelor of Medicine and Bachelor of Surgery (MBBS) batch alumnus of Nil Ratan Sircar Medical College, Kolkata [3]. He provided the Jarawa people with health care and protection [4]. For his efforts to prevent the extinction of the Jarawa tribe, he received the Padma Shri in the medical field in March 2023 [2].

He held various designations, such as executive secretary, chief medical officer, deputy director of tribal health, chief medical officer in charge, and more. He assumed responsibility for the Konyak tribe's medical requirements when he started working for the Nagaland Department of Health & Family Welfare. After retiring, he spent seven years working for the National Rural Health Mission in the Andaman and Nicobar Islands. He eventually relocated to the Directorate of Health Services for the Andaman and Nicobar Islands [2].

## Review

The Jarawa tribes of Andaman and Nicobar Islands

The enchanting archipelago of Andaman and Nicobar Islands is a treasure of biodiversity and the antiquity of human evolution, which dates back to 2000 years. There are six tribal groups on these islands, of which five are identified as primitive tribes [5].

The Jarawas, one of the primitive tribes, are an indigenous people of the Andaman Islands. They live in parts of Middle and South Andaman. Contacts between outsiders and Jarawa communities have increased in frequency since the 1990s [6]. 

But as the Jarawas started mingling with outsiders, there began epidemics of diseases like measles, mumps, and hepatitis E that their bodies had never developed immunity against. Dr. Kar calls these the gift of civilization. These outbreaks nearly wiped out their population [7].

Dr. Kar discovered the Jarawa to be in remarkably good condition when he first visited them in 1998. According to him, they are even better than the average rural people, well-built, and apparently healthier than any other tribal community on the mainland.

Conditions like hypertension, obesity, mental illness, and heart disease were foreign to them, and they were practically disease-free. Skin sores, worm infestations, and injuries from crocodile attacks were prevalent diseases. Every family made "alam," which was a red clay powder combined with pig fat and applied to mild aches and pains [8].

An event took place in April of 1996. One night, a neighboring settlement found a Jarawa youngster named Enmai who slipped and broke his leg. The settlers brought him to Port Blair Hospital, where he received two months of treatment. Upon his recovery, he was brought back to the Jarawas. For those two months, his people believed Enmai had passed away. He told the Jarawas how he was cared for after being sent home. After hearing his narrative, the Jarawa resolved not to use a bow and arrow on strangers. They began socializing with others around the end of 1997 and the beginning of 1998. They began catching infectious illnesses like measles after they started interacting with civilized people. Numerous people were dying [4].

The measles outbreak of 1999

September and October of 1999 saw a serious measles outbreak on the islands. It was believed by the Jarawa people that they would go extinct during this measles epidemic [2]. It was known that 108 Jarawa had contracted measles during this outbreak. They are very susceptible, much like many tribal people who are rarely contacted. Measles has wiped out a large number of tribal people. It eliminated all the Great Andamanese on one island and at least half of them on another in the 19th century. From 5,000 members, that tribe currently consists of only 41 individuals [9].

Dr. Kar’s role in the 1999 measles epidemic

To protect the tribe from measles, the welfare workers who were visiting them asked the center to assign a doctor to live among them. When the central government ran advertisements, there was very little reaction. However, because Dr. Kar had previously worked in Nagaland with the Konyak tribe, he showed his interest in working with the Jarawa tribe. He was chosen by the central government to work at Kadamtala Hospital in the central Andaman Islands in 1999. The Jarawa tribe resided in a dense, impenetrable jungle near the Kadamtala hospital [4]. The Jarawa tribes often look at outsiders with suspicion and are territorial. They were aggressive and armed with poisonous arrows [10]. Tribal people were succumbing to measles. Dr. Kar accepted the challenge and committed himself to the mission of eradicating the disease [4]. He was eventually able to win the trust of the tribal community and provide them with medical help in the most critical time [10].

**Figure 2:**
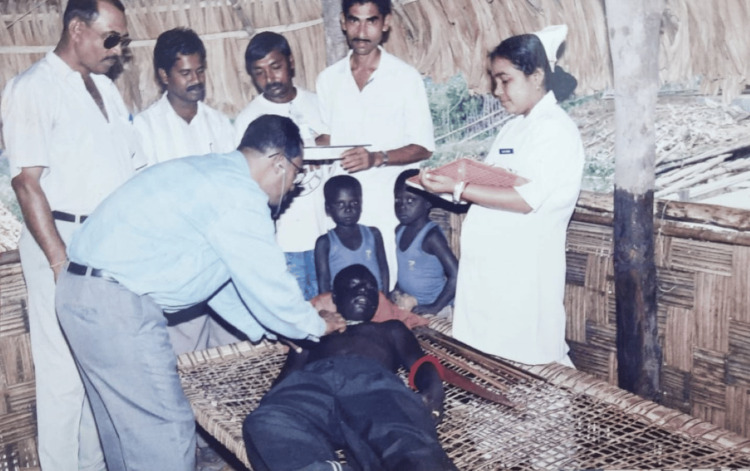
Dr. Kar examining a Jarawa patient Image courtesy: Dr. Ratan Chandra Kar Source: [4]

The government also pledged full support because, throughout the world, there was concern that the Jarawas would face extinction [4]. He became familiar with their culture and mastered their language in four to five months. In an attempt to make friends, he went door to door. The Jarawa people have a 50,000-year-old traditional medical system. Dr. Kar was impressed by their therapeutic approach and saw firsthand how they used natural medications. Nevertheless, he and his team used contemporary medications as life-saving interventions (Figure 2). Respecting their traditional medicines, he employed his own as a supplemental treatment [3].

To receive medical attention, they occasionally swam between the islands and hiked for kilometers through the wilderness. Although he respected their way of life, he also persuaded them that they needed to be admitted to hospitals in case something severe occurred [11]. Every day, he provided medication and assessed 15 to 20 patients. He saw up to 40 cases a day during the peak of the measles outbreak. He transported seriously ill individuals from the jungle to the hospital as needed.

Dr. Kar built a Jarawa Ward, an environmentally friendly building with a floor made of concrete, at Kadamtala Hospital. Bamboo, hay, and dried tree trunks were used to create a setting that mirrored their homes. They constructed a communal bathroom with a six-person shower and a fireplace in the center of the ward. Only Dr. Kar’s personnel were permitted entry into the restricted area, which was manned by police around the clock. The tribal members initially misinterpreted the area and believed it to be a prison, but eventually, they began to feel comfortable. Ultimately, the tribes were spared extinction [2]. Serving for the Jarawa people became his life's purpose. He was disappointed that no one else appeared to be concerned about their welfare. The tribe was disregarded since most people thought they were a dangerous ethnic group. Thus, Dr. Kar played a crucial role in combating the measles epidemic among the Jarawas. “The outbreak took place in 1999 between September and October. The Jarawas had started to trust me by then. I checked over a hundred of them into a nearby hospital. Everyone who visited the hospital was healed and released,” Dr. Kar said [11].

Vaccination

Regarding the vaccination debate, Dr. Kar advised prudence. He contended that the Jarawa people's "inherent immunity" was superior to "immunity gained, if at all, done by modern vaccination system." The absence of hepatitis B, even though over half of the population carried the virus, is one indication of their immunity. The fact that more than 50% of the Jarawa tested positive for the hepatitis B antigen, probably the highest ever reported in the world, concerned other scientists. Nowadays, immunization is a must for all Jarawa kids [8].

 

Padma Shri 2023

**Figure 3:**
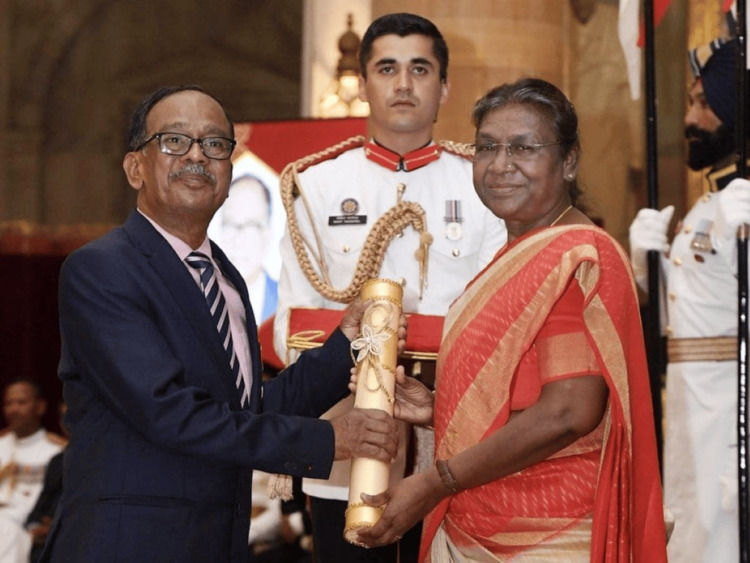
Dr. Ratan Chandra Kar receiving the Padma Shri award from the honorable President of India, Smt. Droupadi Murmu in year 2023 Image courtesy: Dr. Ratan Chandra Kar Source: [4]

The honorable President of India, Smt. Droupadi Murmu awarded Dr. Kar the Padma Shri (Figure 3) in recognition of his unwavering commitment to providing medical care to the Jarawas, who live on an island almost 50 kilometers from the North Sentinel of the Andaman. The award was also a token of appreciation for the significant contribution of Dr. Kar during the measles outbreak in 1998-1999 and his crucial actions in preventing the Jarawa community from going extinct [10].

## Conclusions

Dr. Kar has provided treatment to members of the Jarawa tribe, who rarely permitted outsiders to enter their territory, and profoundly impacted their health and well-being. They allowed Dr. Kar to enter their settlement area, especially during the measles outbreak of 1999, which highlights the importance of culturally sensitive healthcare approaches in tribes. Dr. Kar received the Padma Shri in the field of medicine in the year 2023 for his unwavering medical assistance to the Jarawas and for saving their lives. Dr. Kar’s cultural sensitivity, combined with medical intervention, served as a model for addressing health challenges in vulnerable and isolated populations.
